# Systematic characterization of *Brassica napus UBC13* genes involved in DNA-damage response and K63-linked polyubiquitination

**DOI:** 10.1186/s12870-023-04035-y

**Published:** 2023-01-12

**Authors:** Ivanthi Kumasaruge, Rui Wen, Lipu Wang, Peng Gao, Gary Peng, Wei Xiao

**Affiliations:** 1 Department of Biochemistry, Microbiology and Immunology, University of Saskatchewan, Saskatoon, SK S7N 5E5 Canada; 2 Saskatoon Research and Development Centre, Agriculture and Agri-Food Canada, Saskatoon, SK S7N 0X2 Canada; 3 Department of Plant Sciences, University of Saskatchewan, Saskatoon, SK S7N 5A8 Canada

**Keywords:** *Brassica napus*, DNA-damage tolerance, K63-linked polyubiquitination, Protein-protein interaction, Abiotic response

## Abstract

**Background:**

Ubc13 is the only known ubiquitin conjugating enzyme (Ubc/E2) dedicated to promoting Lys (K)63-linked polyubiquitination, and this process requires a Ubc/E2 variant (UEV). Unlike conventional K48-linked polyubiquitination that targets proteins for degradation, K63-linked polyubiquitination, which is involved in several cellular processes, does not target proteins for degradation but alter their activities.

**Results:**

In this study we report the identification and functional characterization of 12 *Brassica napus UBC13* genes. All the cloned *UBC13* gene products were able to physically interact with AtUev1D, an *Arabidopsis* UEV, to form stable complexes that are capable of catalyzing K63-linked polyubiquitination in vitro. Furthermore, *BnUBC13* genes functionally complemented the yeast *ubc13* null mutant defects in spontaneous mutagenesis and DNA-damage responses, suggesting that *BnUBC13*s can replace yeast *UBC13* in mediating K63-linked polyubiquitination and error-free DNA-damage tolerance.

**Conclusion:**

Collectively, this study provides convincing data to support notions that *B. napus* Ubc13s promote K63-linked polyubiquitination and are probably required for abiotic stress response. Since plant Ubc13-UEV are also implicated in other developmental and stress responses, this systematic study sets a milestone in exploring roles of K63-linked polyubiquitination in this agriculturally important crop.

**Supplementary Information:**

The online version contains supplementary material available at 10.1186/s12870-023-04035-y.

## Background

Ubiquitin (Ub) is an abundant and one of most highly conserved proteins in eukaryotes, from unicellular yeast to human. Ub is able to conjugate with other proteins by forming an isopeptide bond using its C-terminal glycine (G76) residue and eventually produces a monoubiquitinated substrate [1]. Ub has two key features. One is that its G76 can form an isopeptide bond with a substrate lysine (K) residue. Another is that seven lysine residues in Ub, namely K6, K11, K27, K29, K33, K48 and K63 can be potentially used to form distinct types of poly-Ub chains; at least five of which have been observed in vitro or in vivo [2].

Ubiquitination, a process of Ub attachment to targeted proteins, regulates diverse cellular activities such as proteasomal and lysosomal degradation, subcellular localization [3], DNA damage response [4, 5], ribosomal biogenesis [6], cell cycle progression [7], apoptosis [8], mitochondrial inheritance [9] and transcriptional regulation [10]. Ub conjugated to the target protein can alter the protein stability, localization or activity [11]. The ubiquitination requires three basic enzymatic activities to work in concert to transfer Ub to client substrates and form poly-Ub chains. Firstly, high energy thioester bond is formed with the C terminus of Ub by a Ub activating enzyme (Uba or E1). The Ub molecule is then transferred to the active-site Cys of a Ub-conjugating enzyme (Ubc or E2). The subsequent transfer of Ub to the ε-amino group of a Lys side chain in the substrate is catalyzed by the Ub-charged E2 after binding to a Ub ligase (E3). It is well established that distinct structural and functional information is conveyed by poly-Ub chains bearing different linkages, in which K48-linked Ub chains mainly target protein for degradation by the 26 S proteosome. In contrast, K63-linked chains confer non-proteolytic functions involved in well-characterized pathways including DNA damage response (DDR) [12, 13], NF-κB activation [14, 15], mitochondrial inheritance [9], plasma membrane protein endocytosis [16], ribosome function [17] and cell-cycle checkpoints [18], in which target proteins conjugated with K63-linked poly-Ub chains affect protein-protein interaction and/or protein localization [1]. Thus far, Ubc13 is the only known E2 specialized in catalyzing K63-linked polyubiquitination, and this activity absolutely requires a Ubc/E2 variant (UEV) as a cofactor [12, 19]. It is well established that K48- and K63-linked poly-Ub chains represent two distinct conformations, in which the 26 S proteosome can only recognize the K48-linked zig-zag conformation, whereas proteins containing specialized tandem Ub-binding domains can recognize K63-linked stretched Ub chains [1, 20].

The model plant *Arabidopsis thaliana* contains two highly conserved *UBC13* genes capable of functionally complementing the yeast *ubc13* null mutant in terms of spontaneous mutagenesis and sensitivity to DNA-damaging agents [21]. Both AtUbc13s can interact with yeast and human UEVs [21] as well as *Arabidopsis* UEVs [22]. Subsequent studies reveal that *AtUBC13*s function in apical dominance [23], DDR [22], iron metabolism [24], auxin signaling [25], low temperature stress response [26] and plant immunity [26, 27]. In addition, systematic studies in *Arabidopsis* revealed additional target proteins modified by K63-linked polyubiquitination [28, 29], indicating that plant Ubc13s play multiple roles in plant development and stress responses.

*Arabidopsis* belongs to the *Brassicaceae* family, which includes large number of economic crops like rapeseed (*Brassica napus*) and its value-added breeding derivative canola [30]. Given the involvement of Ubc13 and K63-linked polyubiquitination in agriculturally related traits, we wish to investigate *B. napus UBC13* genes. Here we report molecular cloning and functional characterization of *UBC13* genes from *B. napus*, following strategies as outlined in Supplementary Fig. S1. All analyzed BnUbc13s can interact with AtUev1D and catalyze K63-linked poly-Ub chain assembly. Since *UBC13* genes have been shown to play critical roles in plant development and responses to both abiotic and biotic stresses including agriculturally important features, this study sets a corner stone in improving the oilseed crop through genetic manipulations.

## Results

### Identification and bioinformatics analyses of *BrassicanapusUBC13* genes

**Fig. 1:**
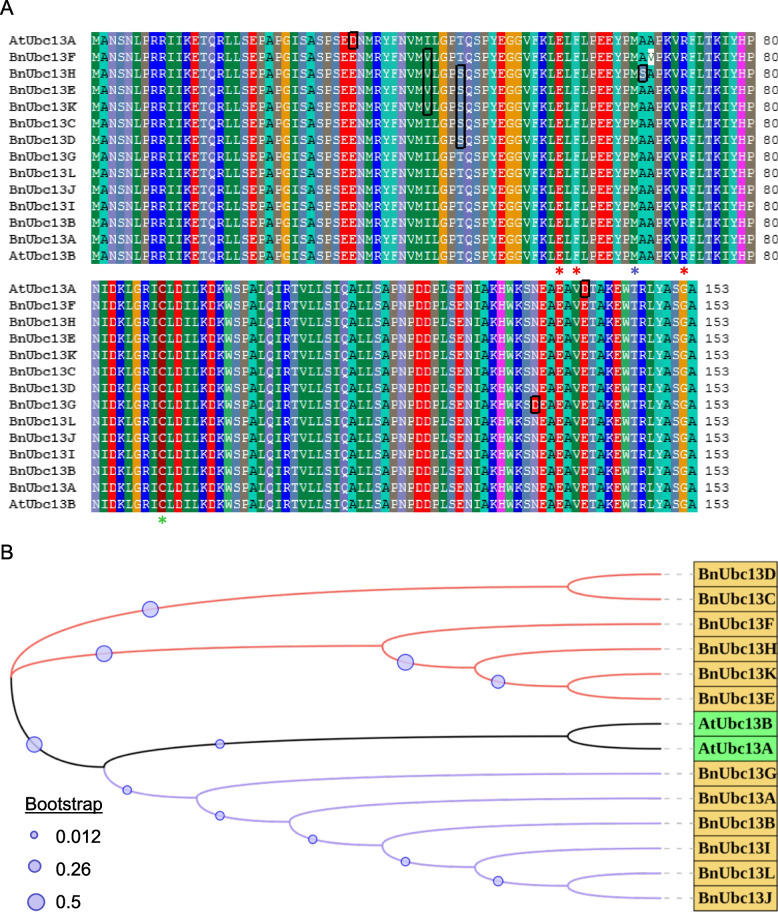
Sequence analysis of Ubc13s from *B. napus* (Bn) and *A. thaliana* (At). **A** Predicted amino acid sequences of *UBC13* gene products from *B. napus* and *Arabidopsis* were aligned by the BioEdit software version 7.2.5. Identical residues shared by the majority of Ubc13s are highlighted. Amino acid residues required for the interaction with UEV are marked by red asterisks, required for the interaction with a RING E3 is marked by a blue asterisk, and the active site Cys residue is marked by a green asterisk. **B** A phylogenetic tree based on *B. napus* and *Arabidopsis* Ubc13 family amino acid sequences was constructed by using MEGA7.0.26 and iTOL. Lines with different colors represent different branches: black, *Arabidopsis*; red and purple, two separate branches of *B. napus*. Yellow and green rectangles highlight Ubc13 proteins from *B. napus* and *Arabidopsis*, respectively. Purple circles indicate bootstrap levels as indicated

To identify *B. napus UBC13* genes, the *Arabidopsis* Ubc13 protein sequence was used to Blast the *B. napus* protein database (https://plants.ensembl.org/Brassica_napus/Tools/Blast). Eight predicted highly similar proteins (E-values at or below 10^− 56^), namely BnaA06g11360D (GenBank accession OP380669), BnaC08g38130D (OP380670), BnaAnng13030D (OP380671), BnaC08g17090D (OP380672), BnaA07g34450D (OP380673), BnaA08g23450D (OP380674), BnaC05g12900D (OP380675) and BnaC06g39290D (OP380676), were found and named as BnUbc13A-H, respectively. In addition, four incomplete proteins in the database, BnaC02g25260D (OP380677), BnaA07g38410D (OP380679), BnaC06g20310D (OP380678) and BnaA02g19070D (OP380680), have about 99% sequence identity with AtUbc13. After cDNA amplification and sequencing, these four encoded proteins were also found to be highly homologous to AtUbc13 and subsequently named BnUbc13I-L. Features of the 12 identified *BnUBC13* genes and gene products, including number of exons, gene location, protein domain and length, molecular weight (MW), isoelectric point (pI) and grand average of hydropathicity index (GRAVY), are summarized in Supplementary Table S1. All 12 BnUbc13 proteins contain 153 amino acids and their alignment with AtUbc13s is shown in Fig. 1A. AtUbc13A and AtUbc13B differ by only two conserved amino acids [21]. Within BnUbc13s, BnUbc13A, B, I, J and L are identical to AtUbc13B; BnUbc13C and D are identical to each other but differ from AtUbc13B by one amino acid; BnUbc13E and K are identical to each other but differ from AtUbc13B by two amino acids; BnUbc13G, F and H differs from AtUbc13B by 1, 2 and 3 amino acids, respectively. These amino acid substitutions do not affect known functional residues, including M66 (blue asterisk) involved in physical interaction with E3s [31], E57, F59 and R72 [32] required for the interaction with UEV, and the active site C89 to conjugate Ub [12]. All BnUbc13s appear to be closer to AtUbc13B in sequence than to AtUbc13A, as the two amino acid variations in AtUbc13A were not found in any BnUbc13s (Fig. 1A).

The phylogenetic analysis of BnUbc13s to AtUbc13s was performed. As shown in Fig. 1B, all 12 BnUbc13s can be grouped into two clades separately derived from AtUbc13A and AtUbc13B, and the bootstraps in the clade of AtUbc13B are higher than that of AtUbc13A. To further assess the conservation of *BnUBC13* genes in *Brassicaceae*, we performed synteny analysis among four related species: *A. thaliana*, *B. napus*, *B. rapa* and *B. oleracea*. As anticipated, the *B. napus* genome was split into two subgenomes A and C, corresponding to *B. rapa* and *B. oleracea* genomes, respectively (Supplementary Fig. S2).

Due to amino acid sequence redundancy, eight *BnUBC13* genes (A to H) representing all BnUbc13 variants were used for the subsequent studies.

### Functional complementation of yeast *ubc13* null mutants by *BnUBC13s*

**Fig. 2:**
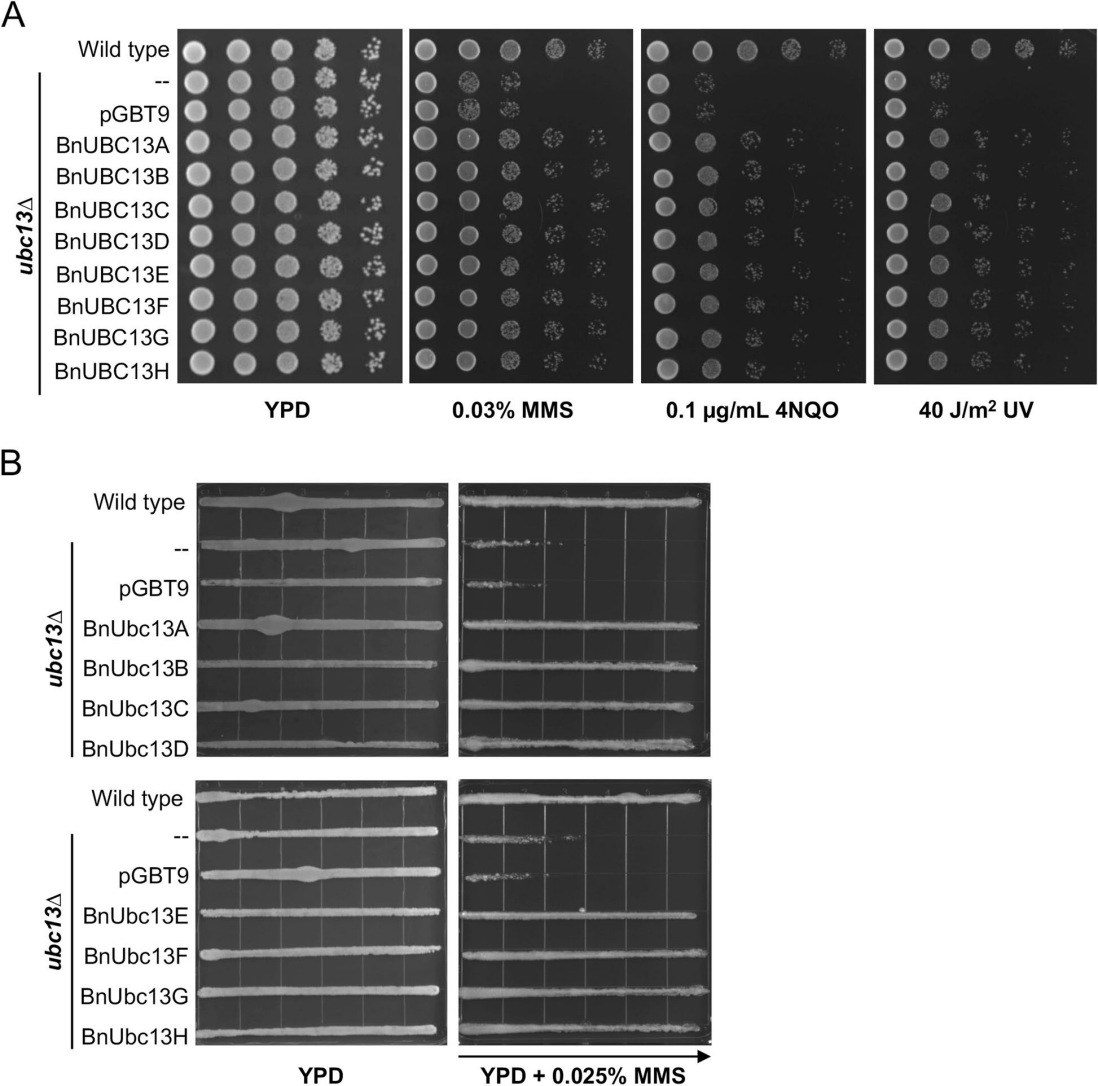
Functional complementation of the yeast *ubc13* null mutant by *BnUBC13s.* WXY904 (*ubc13∆*) cells transformed with vector pGBT9E or plasmids carrying indicated *BnUBC13* genes were grown in the selective medium overnight and the cell density was adjusted. **A** A serial dilution assay. Cells were diluted and printed onto YPD plates with or without different concentrations of 4NQO or MMS. For the UV irradiation, plates containing printed cells were exposed to 254 nm UV at given doses. **B** A gradient plate assay. Cells were printed to a square plate across the MMS gradient at given concentrations. The plates were incubated at 30 ºC for 2 days before being photographed. The arrow points to increasing MMS concentrations. Several doses of DNA-damaging agents were examined and only one representative plate is shown. For each sample, several independent colonies from each transformation were examined with comparable results, and only one set of plates is shown. Wild type, HK578-10D.

Budding yeast *UBC13* functions in the error-free DNA-damage tolerance (DDT) pathway [33–35]. To ask whether *BnUBC13*s have same functions as yeast *UBC13*, pGBT-BnUBC13 plasmids were transformed into the yeast *ubc13∆* mutant and cell survival in the presence of various DNA-damaging agents were examined by a serial dilution assay. As previously reported [34], the yeast *ubc13* mutant displayed an increased sensitivity to a variety of DNA-damaging agents including methyl methanesulfonate (MMS), 4-nitroquinoline 1-oxide (4NQO) and ultraviolet (UV) irradiation, while expression of *BnUBC13s* could functionally complement the yeast *ubc13* DDT defect (Fig. 2A). Similarly, in a gradient plate assay, deletion of *UBC13* resulted in an increased sensitivity to MMS, while pGBT-BnUBC13s, but not the pGBT9 empty vector, were able to restore cellular resistance to MMS (Fig. 2B).

**Fig. 3:**
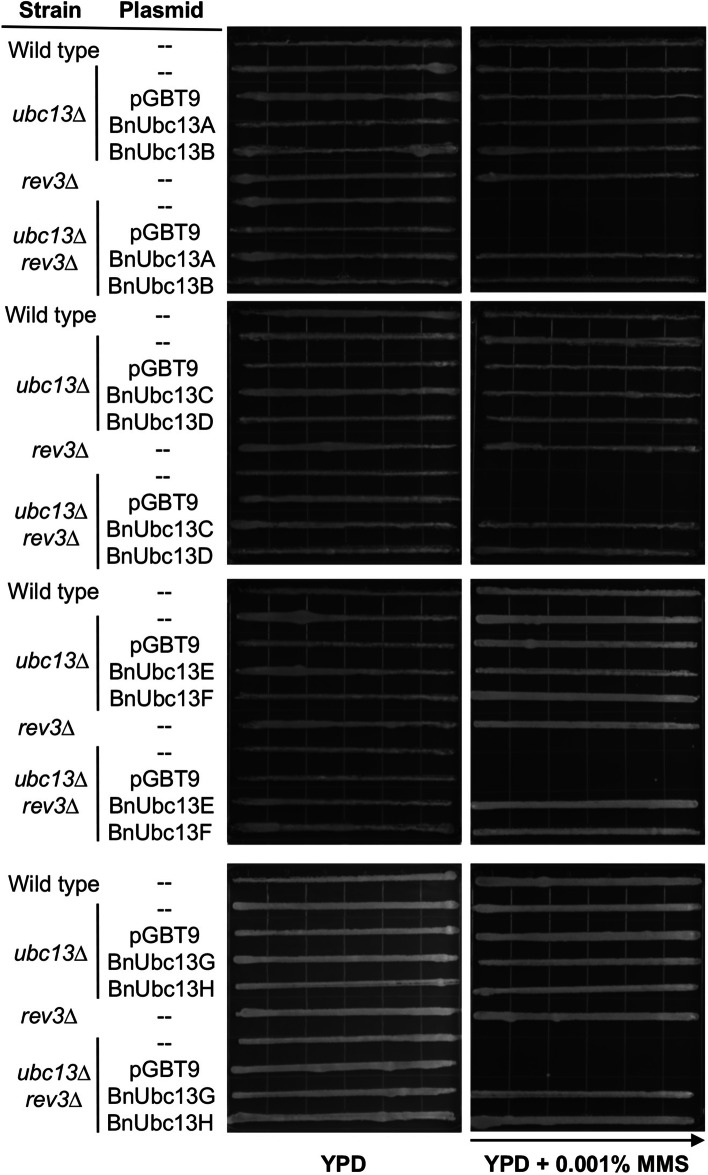
Functional complementation of the yeast *ubc13 rev3* double mutant by *BnUBC13s.* WXY904 (*ubc13*Δ) and WXY921 (*ubc13Δ rev3Δ*) cells transformed with vector pGBT9E or plasmids carrying indicated *BnUBC13* genes were grown in the selective medium overnight. After adjustment of the cell density, cells were printed onto plates containing different concentrations of MMS gradient, and the plates were incubated at 30 ºC for 2 days before being photographed. The arrow points to increasing MMS concentrations. Several independent colonies from each transformation were examined with comparable results, and only one set of plates is shown here. Wild type, HK578-10D; *rev3Δ*, WXY1233.

Yeast *UBC13* functions in an error-free DDT pathway in parallel to the translesion DNA synthesis (TLS) pathway in response to lesions that block replication [34]. While mutations in each pathway cause moderate sensitivity to DNA damaging agents, double mutations defective in both pathways result in synergistic effects [36]. Figure 3 shows that in the presence of 0.001% MMS, neither *ubc13*∆ nor *rev3*∆ single mutants displayed increased sensitivity; however, the *ubc13∆ rev3*∆ double mutant did not grow at all in the gradient plat assay. Under the same experimental conditions, expression of any of the eight *GAL4*_*BD*_*-BnUBC13* genes, but not *GAL4*_*BD*_ alone, was able to rescue the double mutant to the level indistinguishable from the wild-type or single mutants (Fig. 3). The above observations collectively demonstrate that *B. napus UBC13* genes can functionally complement the yeast *ubc13*∆ mutant from killing by DNA-damaging agents.

### *BnUBC13* genes protect *ubc13* cells from spontaneous mutagenesis

**Fig. 4:**
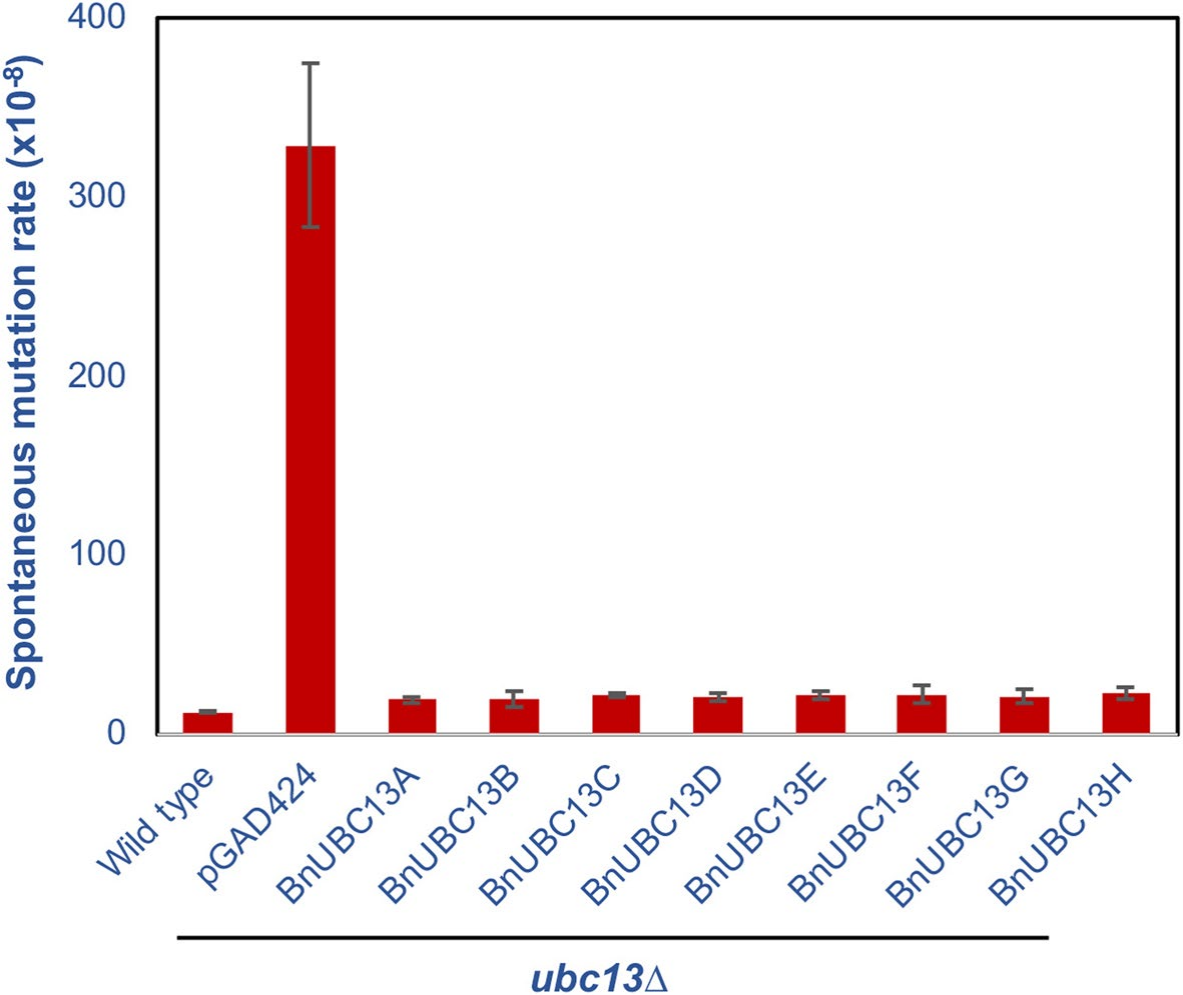
Effects of *BnUBC13*s on spontaneous mutagenesis. WXY849 (*ubc13*∆) cells transformed with vector pGAD424E or plasmids carrying *BnUBC13* genes were grown in the selective medium, diluted in YPD, incubated at 30 ^o^C for 3 days, plated onto SD-Leu and SD-Leu-Trp, and the plates were incubated for 3 days before counting number of colonies. Spontaneous mutation rates were calculated [54] and presented as mutation events per cell per generation. Wild type, DBY747

Yeast *UBC13* is a member of the error-free DDT pathway and plays an important role in protecting yeast cells from spontaneous mutagenesis [33, 34]. Therefore, a spontaneous mutagenesis assay was performed to determine whether *BnUBC13s* could functionally complement the error-free DDT defect in the yeast. It was apparent from Fig. 4 that inactivation of *UBC13* in wild-type yeast cells caused nearly 27-fold increase in the spontaneous mutation rate. The large increase in spontaneous mutagenesis supports a notion that *UBC13* plays a vital role in maintaining host genome stability. In contrast, when *ubc13*Δs cells were transformed with *BnUBC13*s, the spontaneous mutation rate dropped to near wild-type levels. These results indicate that BnUbc13s can replace Ubc13 in yeast cells to limit endogenous DNA-damage stress.

### Physical interactions of BnUbc13s with AtUev1D

**Fig. 5:**
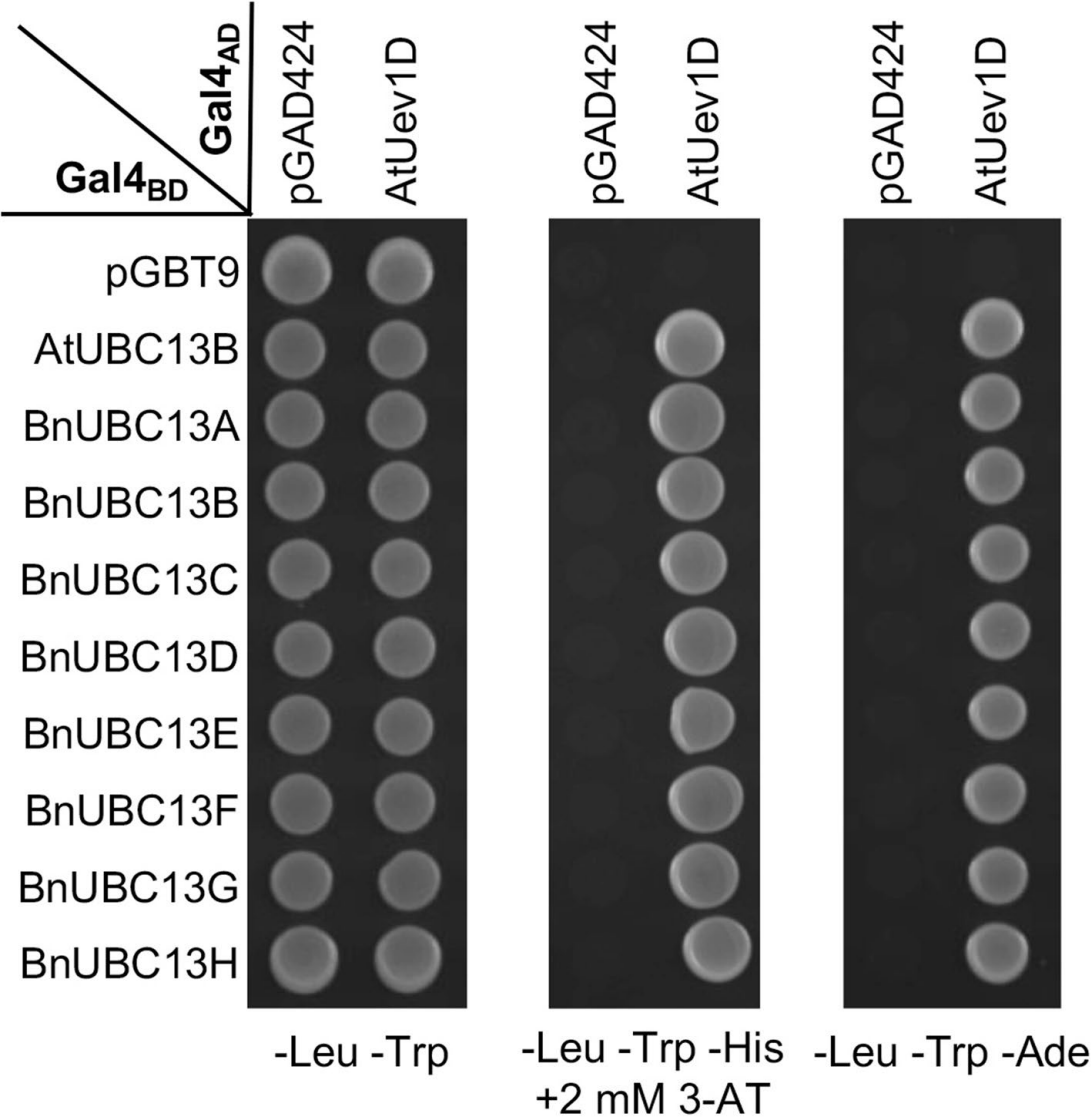
Physical interactions between BnUbc13s and AtUev1D in a yeast two-hybrid assay. PJ69-4 A cells co-transformed with vectors pGBT9E (Gal4_BD_) and pGAD424E (Gal4_AD_) or plasmids carrying *AtUEV1D* and indicated *BnUBC13*s were grown in the SD-Leu-Trp medium overnight. After adjustment of cell density, cells were replicated on SD-Trp-Leu (control), SD-Trp-Leu-His plus various concentrations of 3-AT, and SD-Trp-Leu-Ade, followed by incubation for 3 days at 30 ^o^C before being photographed. Five independent colonies from each transformation were examined with comparable results, and only one set is shown here

One condition for the yeast Ubc13 function is that it has to form a stable complex with Mms2, the only known UEV in yeast cells [12]. The fact that BnUbc13s can replace yeast Ubc13 in spontaneous and DNA-damage induced functions indicates that it must interact with Mms2 in yeast cells. To ask whether BnUbc13s are also able to interact with plant UEVs, a yeast two hybrid (Y2H) assay [37] was employed to analyze the protein-protein interaction between BnUbc13s and AtUev1D. All eight Gal4_BD_-BnUbc13s gave positive results with Gal4_AD_-AtUev1D under high stringency (SD-His + 3-AT and SD-Ade) conditions in comparison to negative controls including Gal4_BD_-BnUbc13s with Gal4_AD_ and Gal4_BD_ with Gal4_AD_-AtUev1D (Fig. 5). All the above interactions are deemed robust and strong, and no difference in the interaction strength among BnUbc13s was observed. Indeed, these interactions seem to be specific between BnUbc13s and AtUev1D as neither of the proteins alone was able to activate reporter genes in the Y2H assay. Therefore, results from the Y2H assay indicate that all BnUbc13s are able to physically interact with AtUev1D.

**Fig. 6:**
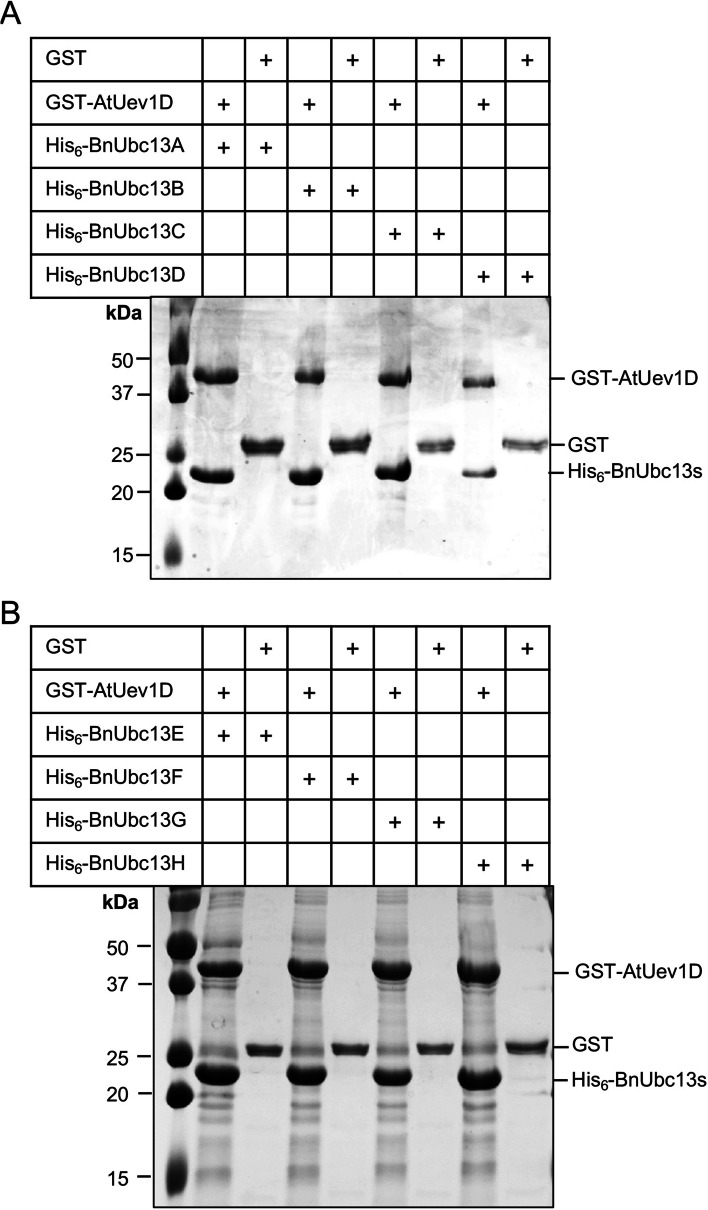
Physical interactions between BnUbc13s and AtUev1D in a GST pulldown assay. Co-purified GST-AtUev1D and His_6_-BnUbc13s were added to microspin columns. After incubation and washing, the columns were eluted with reduced glutathione and subjected to SDS-PAGE analysis. **A** AtUev1D with BnUbc13A-D. **B** AtUev1D with BnUbc13E-H. Identities of key bands are marked. Unprocessed WB images shown in A, B are given in Supplementary Fig. S3A, B

The physical interaction between BnUbc13 and AtUev1D was further confirmed independently in vitro by a GST-affinity pulldown assay. In this experiment, bacterial cells were transformed with both His_6_-tagged BnUbc13s and GST-tagged AtUev1D, and the produced proteins in bacterial cells were co-purified by adding to a column containing glutathione beads. After incubation, washing and elution, all eight His_6_-BnUbc13s were found to be co-eluted with GST-AtUev1D, but not with GST (Fig. 6). Hence, all BnUbc13s can form stable heterodimers with AtUev1D in vitro.

### Dual rescue of yeast *ubc13 mms2* by *BnUBC13s* and *AtUEV1D*

**Fig. 7:**
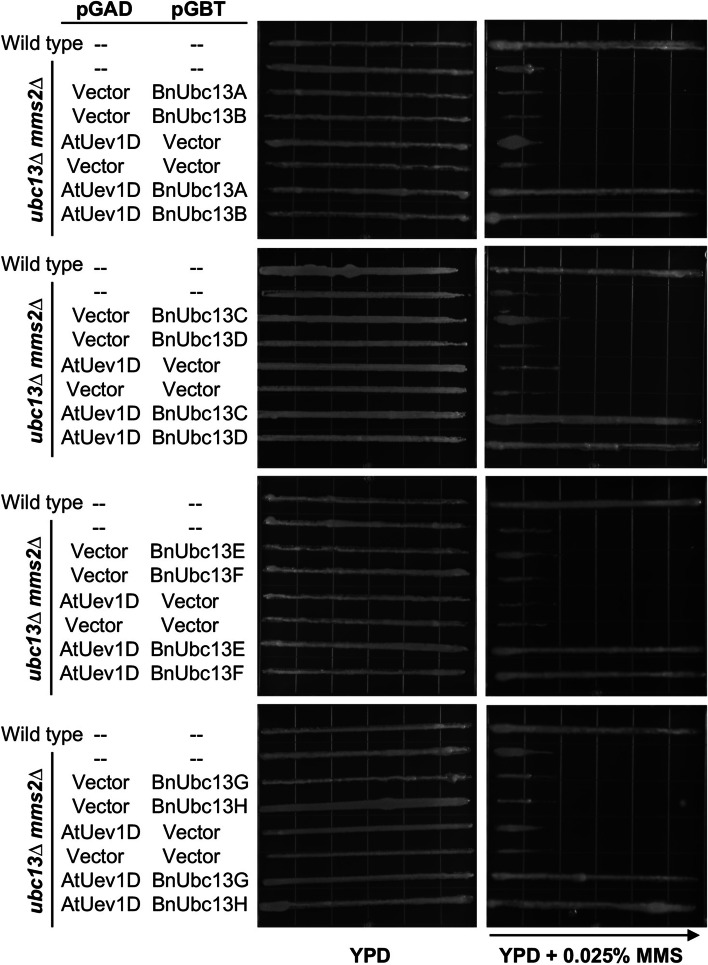
Functional complementation of the yeast *ubc13 mms2* double mutant by *BnUBC13*s and *AtUEV1D*. WXY955 (*ubc13Δ mms2Δ*) cells co-transformed with vectors pGBT9E and pGAD424E or with plasmids containing *AtUEV1D* and indicated *BnUBC13*s were grown in the SD-Leu-Trp selective medium overnight. After adjustment with cell density, cells were printed onto YPD plates containing 0.025% MMS gradient and the plates were incubated at 30 ºC for 2 days before being photographed. The arrow points to increasing MMS concentrations. Several independent colonies from each transformation were examined with comparable results, and only one set of plates is shown here. Wild type, HK578-10D.

To assess in vivo complex formation and functions between BnUbc13s and AtUev1D, the *ubc13 mms2* double mutant was created and co-transformed with pGBT-BnUBC13s and pGAD-AtUEV1D, or with their respective empty vectors. When the double mutant cells were transformed with only pGBT-BnUBC13s or pGAD-AtUEV1D, the transformed cells did not display enhanced resistance to MMS (Fig. 7), implying that both Ubc13 and UEV are required for the DDT function. In contrast, double mutant cells carrying both pGBT-BnUBC13 and pGAD-AtUEV1D plasmids displayed MMS resistance comparable to the wild-type level (Fig. 7). Since *BnUBC13*s and *AtUEV1D* can jointly complement *ubc13*Δ and *mms2*Δ defects in yeast, one can envisage that BnUbc13s must be able to bind AtUev1D in yeast cells to form a functional E2 complex and promote K63-linked polyubiquitination on PCNA [13], which is a highly conserved process within eukaryotes including plants [38].

### BnUbc13 mediates K63-linked polyubiquitination with AtUev1 *in vitro*

**Fig. 8:**
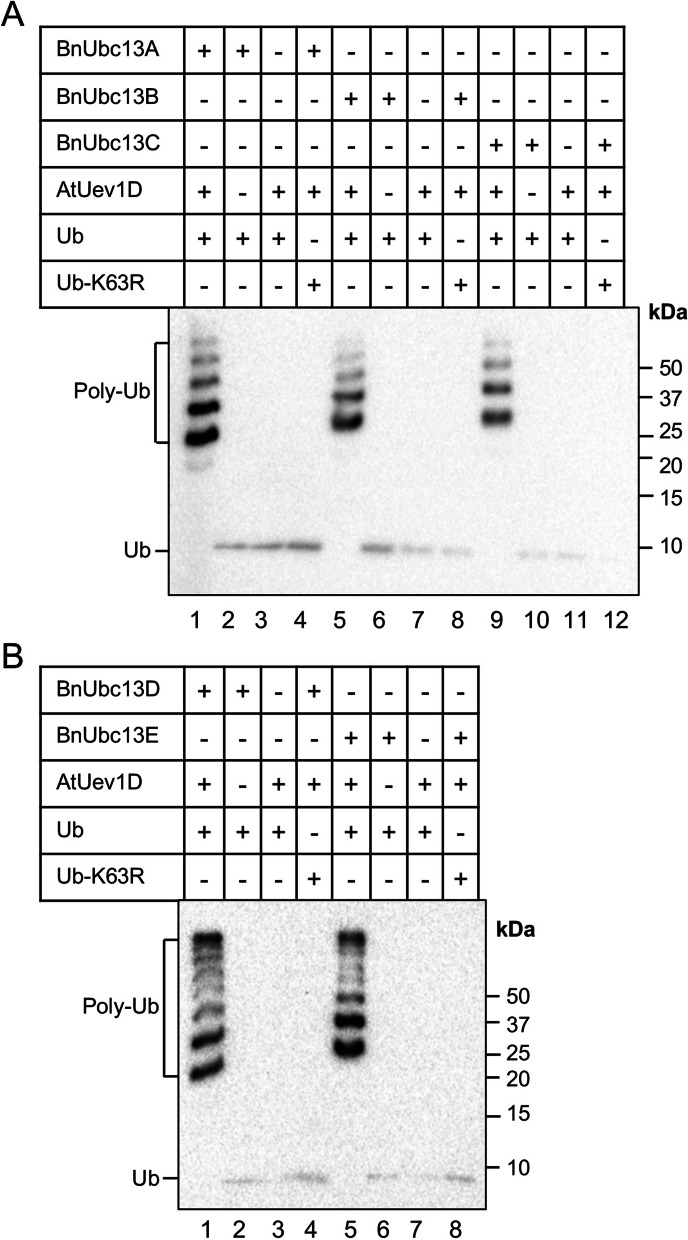
The in vitro Ub conjugation assay using purified BnUbc13s and AtUev1D. **A** Ub conjugation by BnUbc13A, B, C and AtUev1D. **B** Ub conjugation by BnUbc13D, E and AtUev1D. An in vitro Ub conjugation assay was performed using purified proteins as indicated. Assay samples were subjected to SDS-PAGE and western blotting analyses using an anti-Ub antibody. Free Ub and poly-Ub chains are marked. Unprocessed WB images shown in **A, B** are given in Supplementary Fig. S4A, B

To directly examine whether BnUbc13s can promote K63-linked poly-Ub chain assembly, we performed an in vitro Ub conjugation assay with selected BnUbc13s. To date, Ubc13 is the only known E2 capable of catalyzing K63-linked polyubiquitination; however, Ubc13 alone is insufficient and requires a UEV as a cofactor [12]. As shown in Fig. 8, in our reconstituted Ub conjugation assay, neither BnUbc13s alone (Fig. 8A, lanes 2, 6 and 10; Fig. 8B, lanes 3 and 7) nor AtUev1D alone (Fig. 8A, lanes 3, 7 and 11; and Fig. 8B, lanes 3 and 7) was able to catalyze the poly-Ub chain formation. When both BnUbc13s and AtUev1D are present (Fig. 8A, lanes 1, 5 and 9; Fig. 8B, lanes 1 and 5), free poly-Ub chains are formed. These poly-Ub chains are deemed to be K63-linked, since when Ub was replaced by Ub-K63R, the poly-Ub chain formation was completely abolished (Fig. 8A, lanes 4, 8 and 12; Fig. 8B, lanes 4 and 8). These results demonstrate that BnUbc13s and AtUev1D can jointly form K63-linked poly-Ub chains in vitro.

## Discussion

In this study, we identified 12 *UBC13* genes from the *B. napus* genome. Most non-plant eukaryotic organisms contain only one *UBC13* gene [39]. Characterized diploid plant genomes contain either one [40] or two [21, 41, 42] *UBC13* genes. Hence, it is surprising that the amphidiploid *B. napus* contains 12 *UBC13* genes. Although their DNA sequences vary, the encoded BnUbc13s are highly conserved with no more than 3 amino acid differences. Furthermore, five BnUbc13s are identical in sequence with AtUbc13B, while the two amino acid variations found in AtUbc13A were not shared by any BnUbc13s, supporting a previous speculation through phylogenetic analysis that AtUbc13A was derived from gene duplication after *Arabidopsis* speciation [40]. Similarly, the remaining seven *BnUBC13* genes encoding BnUbc13 variants were likely evolved within the *Brassica* genus. It remains interesting what evolutionary pressure caused sequential *UBC13* gene duplications in *B. rapa* and *B. oleracea*, and then in *B. napus*.

In this study, we cloned *BnUBC13* genes encoding all BnUbc13 variants and characterized their functions in budding yeast. The only known function of yeast *UBC13* to date is its involvement in error-free DDT, which is achieved by promoting K63-linked polyubiquitination of PCNA at the K164 residue [13] that promotes template switch to bypass replication-blocking lesions [35]. This study demonstrated that *BnUBC13*s can functionally complement the yeast *ubc13* null defect in error-free DDT. Firstly, heterologous expression of *BnUBC13*s rescued *ubc13*∆ and *ubc13∆ rev3*∆ cells from killing by DNA-damaging agents, indicating that they function in DDR. Secondly, *BnUBC13*s could also limit the increased spontaneous mutagenesis in *ubc13*∆ cells, suggesting that they protect cells from genome instability under non-stressed conditions. This study also demonstrated physical interactions between BnUbc13s and AtUev1 by Y2H and GST pulldown assays, as well as by a dual rescuing experiment in which BnUbc13s must interact with AtUev1 in yeast cells to restore the DDT function, hence confirming that they form stable heterodimers in vitro and in vivo. Finally, selected BnUbc13 proteins were used in an in vitro Ub conjugation assay, which demonstrated that only in the presence of both BnUbc13 and AtUev1 could free poly-Ub chains be formed, and that these chains were K63 linked. In addition, the dual rescue experiment of yeast *ubc13∆ mms2*∆ mutant cells by *BnUBC13*s and *AtUEV1D* also implies that the BnUbc13-AtUev1D heterodimer must promote PCNA polyubiquitination to confer error-free DDT functions. Although this study did not use a UEV from *B. napus*, the facts that, like Ubc13s, eukaryotic UEVs are also highly conserved and that BnUbc13s could physically interact with both AtUev1 and yeast Mms2 to promote K63-linked polyubiquitination support a notion that *B. napus* Ubc13s and UEVs also form stable heterodimers to promote K63-linked polyubiquitination and are required for plant DDR.

All assays employed in this study did not reveal qualitative or quantitative difference among all BnUbc13s, which is not surprising, since they are highly conserved in sequence with very few amino acid variations and that all amino acid residues with functional implications are identical. Indeed, plant *UBC13* appears to be a housekeeping gene, particularly when the plant genome contains only one *UBC13* gene [40]. However, the similarity in biochemical activity does not necessarily mean that all *BnUBC13* genes function equally and redundantly, as one cannot rule out possibilities that these genes contain different promoter sequences and display different spatial and temporal expression patterns, particularly in response to environmental stresses.

Plant Ubc13-Uev1 complexes are not only required for DDR, but also for other agriculturally related processes. It appears that in multicellular organisms, UEVs serve as regulatory subunits to alter substrate specificity. In human cells, two UEVs, Uev1A and Mms2 contain different N-terminal extension and function in NF-kB activation and DDR, respectively [43]. Plants also contain two classes of Uev1s with different C-terminal extension and cellular activities [44]. We are particularly interested in roles of plant Ubc13 and its mediated K63-linked polyubiquitination in auxin signaling [23, 25, 45], nutrient metabolism [24] and innate immunity against pathogen infections [26, 27, 46], as alteration of these processes may improve quality and yield of the oilseed crop.

## Methods

All methods were performed in accordance with the relevant guidelines and regulations.

### Plant materials and yeast cell culture

*Brassica napus* L. cv Westar was developed at the Saskatoon Research and Development Centre, Agriculture and Agri-Food Canada (AAFC), and registered by AAFC in Canada in 1982 under the registration # 2238. The doubled haploid seed of Westar used in the study was produced at Nutrien Ag Solutions Inc. (Saskatoon, SK, Canada), and provided to AAFC for research uses, including this study. The seeds were planted in Sunshine #3 soil (SunGro Horticulture, Vancouver, BC) in 4-inch pots, and placed in a growth chamber at 22/16°C (day/night) with a 16 h photoperiod until sample collection.

*Saccharomyces cerevisiae* haploid strains used in this study are listed in Supplementary Table S2. Either rich YPD or a synthetic dextrose (SD) medium (0.67% Bacto-yeast nitrogen base without amino acids, 2% glucose) were used to grow yeast cells at 30 ^o^C with necessary nutrients as recommended [47]. When making solid plates, 2% agar was added to either YPD or SD medium prior to autoclaving. Yeast transformation was carried out by dimethyl sulfoxide (DMSO)-enhanced method as described [48].

### Bioinformatics analyses

The genome structure and location of *BnUBC13* genes were retrieved from Ensemble Plants (https://plants.ensembl.org/index.html), illustrating the annotated protein-coding regions. The protein domain was found in the NCBI Conserved Domain Search (https://www.ncbi.nlm.nih.gov/Structure/cdd/wrpsb.cgi), while the subcellular location was predicted by UniProt (https://www.uniprot.org/). The PI and MW were calculated in Expasy (https://web.expasy.org/compute_pi/) by entering the sequences of each gene. The SMS Protein GRAVY software (https://www.bioinformatics.org/sms2/protein_gravy.html) was used to calculate the GRAVY value from the entered FASTA sequences.

A phylogenetic tree based on *B. napus* and *Arabidopsis* Ubc13 family amino acid sequences was constructed by using a maximum likelihood (ML) estimation method of a JTT matrix-based model of MEGA7.0.26. The bootstrap consensus tree inferred from 1,000 replicates. Values < 50% are omitted (parameters: Gaps/Missing data treatment -Partial deletion; Site coverage cutoff − 95%). The phylogenetic tree was exported as Newick format and opened in the Interactive Tree Of Life (iTOL), a web-based tool for phylogenetic tree manipulation and annotation [49].

To identify homologs of *UBC13* family genes and the conservation of these homologous genes in *Brassicaceae*, the synteny analysis was performed among four species: *A. thaliana*, *B. napus*, *B. rapa* and *B. oleracea*. The genome assemblies for these four species were obtained from EnsemblPlants (https://plants.ensembl.org/info/data/ftp, version 55). The synteny analysis was performed by MCScanX [50] with default parameters from top-five BlastP hits. Two *Arabidopsis UBC13* genes (AT1G16890 and AT1G78870) were used to search for gene pairs in three *Brassica* species. When performing the synteny analysis among *Brassica* species, the genome of *B. napus* was split into two subgenomes (A and C), with gene pairs detected between the A subgenome and *B. rapa*, and between the C subgenome and *B. oleracea*, correspondingly. Circos plot for synteny analysis was further performed through http://circos.ca/.

### Cloning *B. napus UBC13* cDNAs and plasmid construction

Total RNA was extracted from *B. napus* seedlings with a TRIzol reagent and used for reverse transcriptase (RT)-PCR with the SuperScript RT-PCR III system (Invitrogen) according to manufacturer’s instructions. Full-length *BnUBC13* coding sequences were cloned from the above resulting cDNA library. All primers used for the PCR are shown in Table S3 and include a *Sal*I restriction site in forward primers and a *Bam*H1 restriction site in reverse primers. The PCR products were cleaved by *Sal*I and *Bam*HI, and subsequently cloned into Y2H vectors pGBT9E and pGAD424E, which were derived from pGBT9 (Gal4_BD_) and pGAD424 (Gal4_AD_) [51], respectively, with a frameshift at the multiple cloning site. The identity of the cloned inserts was confirmed by DNA sequencing.

### Yeast survival assays

A yeast strain HK578-10D and its isogenic mutant derivatives (Table S2) were transformed or co-transformed with pGBT9 and/or pGAD424 based plasmids as indicated, and at least five colonies were selected after 3-day incubation on the selective plate (SD-Leu plates for the single transformation and SD-Leu-Trp for double transformations) and streaked onto the same selective plates. The cell survival assays were as previously described [52]. Briefly, for a gradient plate assay, transformed yeast cells were used to inoculate liquid SD minimal medium. Equal number of cells were taken after overnight incubation and imprinted onto YPD alone or YPD gradient plates containing 0.025% MMS gradient in the bottom layer. 0.1 mL of overnight culture was mixed with 0.4 mL of sterile distilled water and 0.5 mL of molten agar on a sterile microscopic slide and the mixture was printed on the plates across the gradient with another microscopic slide. All the plates were incubated at 30 ^o^C for 3 days before photography.

For a serial dilution assay, overnight cultures were adjusted with equal cell density, made tenfold serial dilutions with sterile distilled water, and 5 µL of each sample was spotted to the plates containing desired concentrations of MMS or 4NQO. For the UV irradiation, samples were spotted to the YPD plates and exposed to predetermined UV irradiation doses in a 254 nM UV crosslinker (UVP). After the liquid was absorbed, the plates were incubated at 30 ^o^C for 3 days before photography.

### Spontaneous mutagenesis assay

A yeast strain DBY747 and its isogenic *ubc13*∆ derivative WXY849 (Table S2) were used in the spontaneous mutagenesis assay. These strains bear a *trp1-289* amber mutation that can be reverted to Trp^+^ by different mutation events [53]. WXY849 was transformed with pGAD-BnUbc13s and the transformants were selected on SD-Leu plates. The overnight yeast cell culture was used to inoculate 5 mL of YPD liquid medium with a final concentration of 20 cells/mL, which was incubated at 30 ^o^C for 3 days. Cells were collected by centrifugation at 4,000 rpm, resuspended in sterile distilled water and plated on SD-Leu to count for the total colony forming units and on SD-Leu-Trp to count for Trp^+^ revertant. Spontaneous mutation rates were calculated as described [54].

### Yeast two-hybrid analysis

The Y2H strain PJ69-4A (Table S2) was co-transformed with Gal4_BD_ and Gal4_AD_ constructs. SD-Leu-Trp plates were used to select the co-transformed colonies. At least five independent colonies from each co-transformation were printed onto SD-Leu-Trp plates and SD-Leu-Trp-His selective plates with different concentrations of 1,2,4 amino triazole (3 A-T) to test activation of the *P*_*GAL1*_*-HIS3* reporter gene and onto Leu-Trp-Ade plates to test activation of the *P*_*GAL2*_*-ADE2* reporter gene. The above plates were incubated at 30 ^o^C for the indicated time before photography.

### Recombinant protein production and extraction

The *BnUBC13* open-reading frames (ORFs) were isolated from pGBT-BnUbc13s and cloned into pET30a (Novogene) as a His_6_ fusion. The resulting pET-BnUbc13s were co-transformed with pGEX-AtUev1D [22] into *Epicurian coli* BL21-CodonPLus (DE3)-RIL strain (Thermo Sci. 2,287,225). The transformed cells were cultured overnight in LB + Amp + Kan, diluted 1:50 into fresh culture and incubated until OD_600 nm_ of 0.6–0.8. The His_6_-BnUbc13s and GST-AtUev1D fusion proteins were induced by adding isopropyl-b-D-thiogalactopyranoside (IPTG) to the final concentration of 0.2 mM and the incubation continued for 6 h. The cells were harvested by 8,000 rpm centrifugation in a Beckman Coulter Avanti JA17 rotor for 1 h at 4 °C, resuspended in phosphate-buffered saline (PBS, 140 mM NaCl, 2.7 mM KCl, 10 mM Na_2_HPO_4_, 1.8 mM KH_2_PO_4_, pH 7.3), and passed through Constant Systems one shot cell disrupter at 25 PSI. The resulting crude extract was centrifuged at 17,000 rpm in the same JA17 rotor for 30 min at 4 ^o^C, and the soluble fraction was used for the GST pulldown assay.

### GST pulldown assay

The GST-pulldown assay was carried out using Glutathione Sepharose 4B Microspin™ GST purification columns as previously described [32]. Purified GST-AtUev1D and His_6_-BnUbc13s were added to individual columns with glutathione beads and incubated for 1 h at 4 ^o^C. Finally, wash buffer (1x PBS buffer with 350 mM NaCl) was used to wash the beads by running through the column. The eluted samples were subjected to 15% SDS-PAGE and visualized by Coomassie blue staining.

### Ub conjugation reaction

*E. coli* BL21-RIL cells separately transformed with pET-BnUbc13s and pGEX-AtUev1D were incubated and induced by IPTG as described above, and the cell extracts were used for protein purification with Bio-Rad poly-prep chromatography columns (731–1550) containing Ni-NTA and glutathione beads, respectively. After washing beads with respective lysis buffers, affinity-purified recombinant proteins were eluted from the columns by using 20 mL of His_6_ elution buffer for His_6_-BnUbc13s and GST elution buffer for GST-AtUev1D. The ubiquitination assay kit containing Ub thioester/conjugation initiation reagents was purchased from Abcam (ab139467). A 20-mL reaction mixture contained E1, Ub, MgATP, reaction buffer from the kit, plus His_6_-BnUbc13 and GST-AtUev1D prepared from this study. The Ub-K63R protein was purchased from Abcam (UM-K63R). Conjugation reactions were performed at 37 °C for 4 h according to manufacturer’s instructions, followed by running of 15% SDS-PAGE and western blotting using polyclonal goat anti-Ub antibodies (Bio-Rad).

## Supplementary Information

**Additional file 1: Table S1.** The characteristics of identified *Brassica napus UBC13* genes and gene products. **Table S2.** *Saccharomyces cerevisiae* strains. **Table S3.** Primers used to amplify *BnUBC13* genes. **Fig. S1.** A flowchart outlining experimental designs of this study. **Fig. S2.** Synteny analysis of *UBC13* family genes among *Arabidopsis thaliana, Brassica napus, B. rapa* and *B. oleracea*. Identified gene pairs were displayed by circos plots (http://circos.ca/). All chromosomes from four species are indicated by blocks scaled by chromosome length with chromosome names. The *UBC13* family genes and chromosomes from different species are indicated by different colors: red, *A. thaliana*; yellow, *B. napus*; purple, *B. rapa* and green, *B. oleracea*. Synteny gene pairs are linked by lines: pink, genes with a close relationship to *AtUBC13A*; brown, genes with a close relationship to *AtUBC13B*. For gene ID from *B. rapa*, the species name “_BraROA” is omitted to reduce the length for plotting. **Fig. S3.** Original images of (A) Fig. 6A and (B) Fig. 6B. **Fig. S4.** Original images of (A) Fig. 8A and (B) Fig. 8B.

## Data Availability

The *BnUBC13* sequences are deposited to the GenBank with accession numbers OP380669-OP380680. The datasets acquired and/or analyzed during the current study are available from the corresponding author on reasonable request.
