# Supplementary material for: Systematic characterization of Brassica napus UBC13 genes involved in DNA-damage response and K63-linked polyubiquitination
Source: BMC Plant Biol. 2023 Jan 12;23:24. doi: 10.1186/s12870-023-04035-y (PMC9835285; doi:10.1186/s12870-023-04035-y)
Supplement: Supplementary file 1 — Additional file 1: Table S1. The characteristics of identified Brassica napus UBC13 genes and gene products. Table S2. Saccharomyces cerevisiae strains. Table S3. Primers used to amplify BnUBC13 genes. Fig. S1. A flowchart outlining experimental designs of this study. Fig. S2. Synteny analysis of UBC13 family genes among Arabidopsis thaliana, Brassica napus, B. rapa and B. oleracea. Identified gene pairs were displayed by circos plots (http://circos.ca/). All chromosomes from four species are indicated by blocks scaled by chromosome length with chromosome names. The UBC13 family genes and chromosomes from different species are indicated by different colors: red, A. thaliana; yellow, B. napus; purple, B. rapa and green, B. oleracea. Synteny gene pairs are linked by lines: pink, genes with a close relationship to AtUBC13A; brown, genes with a close relationship to AtUBC13B. For gene ID from B. rapa, the species name “_BraROA” is omitted to reduce the length for plotting. Fig. S3. Original images of (A) Fig. 6A and (B) Fig. 6B. Fig. S4. Original images of (A) Fig. 8A and (B) Fig. 8B. [file 12870_2023_4035_MOESM1_ESM.docx]

**Supplementary information**

**Table S1**. The characteristics of identified *Brassica napus* *UBC13* genes and gene products

| **Gene Name** | **Gene ID** | **Exon No.** | **Chr** | **Genome location (bp)** | **Protein domain** | **Subcellular localization** | **Protein length** | **MW(Da)** | **pI** | **GRAVY** |
| --- | --- | --- | --- | --- | --- | --- | --- | --- | --- | --- |
| *BnUBC13A* | BnaA06g11360D | 8 | A06 | 344,258-346,977 | Ubc-E2 | Nucleus | 153 | 17219.87 | 6.74 | -0.319 |
| *BnUBC13B* | BnaC08g38130D | 8 | C08 | 785,710-787,683 | Ubc-E2 | Nucleus | 153 | 17219.87 | 6.74 | -0.319 |
| *BnUBC13C* | BnaAnng13030D | 8 | A | 2,809-4,846 | Ubc-E2 | Nucleus | 153 | 17205.85 | 6.74 | -0.320 |
| *BnUBC13D* | BnaC08g17090D | 8 | C08 | 1,325,895-1,327,721 | Ubc-E2 | Nucleus | 153 | 17205.85 | 6.74 | -0.320 |
| *BnUBC13E* | BnaA07g34450D | 8 | A07 | 589,771-591,755 | Ubc-E2 | Nucleus | 153 | 17191.82 | 6.74 | -0.322 |
| *BnUBC13F* | BnaA08g23450D | 8 | A08 | 1,018,769-1,020,653 | Ubc-E2 | Nucleus | 153 | 17233.90 | 6.74 | -0.305 |
| *BnUBC13G* | BnaC05g12900D | 8 | C05 | 157,271-159,450 | Ubc-E2 | Nucleus | 153 | 17220.86 | 6.14 | -0.319 |
| *BnUBC13H* | BnaC06g39290D | 8 | C06 | 1,242,282-1,244,366 | Ubc-E2 | Nucleus | 153 | 17207.82 | 6.74 | -0.339 |
| *BnUBC13I* | BnaC02g25260D | 8 | C02 | 55,248-55,820 | Ubc-E2 | Nucleus | 153 | 17219.87 | 6.74 | -0.319 |
| *BnUBC13J* | BnaA07g38410D | 8 | A07 | 11,368-13,148 | Ubc-E2 | Nucleus | 153 | 17219.87 | 6.74 | -0.319 |
| *BnUBC13K* | BnaC06g20310D | 8 | C06 | 952,275-954,207 | Ubc-E2 | Nucleus | 153 | 17191.82 | 6.74 | -0.322 |
| *BnUBC13L* | BnaA02g19070D | 8 | A02 | 411,743-413,324 | Ubc-E2 | Nucleus | 153 | 17219.87 | 6.74 | -0.319 |

None of the predicted BnUbc13 proteins contains a trans-membrane domain

**Table S2**. *Saccharomyces cerevisiae* strains

| Strain | Genotype | Source |
| --- | --- | --- |
| PJ69-4a | *MAT****a****trp1-901 leu2-3,112 ura3-52 his3-200 gal4Δ gal80Δ MET2::GAL7-lacZ LYS2::GAL1-HIS3 GAL2-ADE2* | P. James |
| HKY580-10D | *MATa ade2-1 can1-100 his3-11,15 leu2-3,112 trp1-1 ura3-1* | H. Klein |
| WXY904 | HKY580-10D with *ubc13∆::HIS3* | Lab stock |
| WXY921 | HKY580-10D with *ubc13∆::HIS3 rev3∆::hisG-URA3-hisG* | Lab stock |
| WXY955 | HKY580-10D with *mms2∆::HIS3* *ubc13∆::hisG-URA3-hisG* | Lab stock |
| WXY1233 | HKY580-10D with *rev3∆::hisG-URA3-hisG* | Lab stock |
| DBY747 | *MAT****a****his3-1 leu2-3,112 trp1-289 ura3-52* | D. Botstein |
| WXY849 | DBY747 with *ubc13Δ*::*HIS3* | Lab stock |

**Table S3**. Primers used to amplify *BnUBC13* genes

| **Primer** | **Sequence (5’- 3’)** |
| --- | --- |
| BnUBC13A-F | CTCGGATCCAAATGGCGAATAGTAATCTTCCT C |
| BnUBC13A-R | CAGTGTCGACTCAAGCGCCACTTGCATATAGAC |
| BnUBC13B-F | CTCGGATCCAAATGGCGAATAGTAATCTACCG |
| BnUBC13B-R | CAGTGTCGACTCAAGCGCCACTTGCATACAGAC |
| BnUBC13C-F | CTCGGATCCAAATGGCGAATAGTAATCTACCG |
| BnUBC13C-R | CAGTGTCGACTCAAGCGCCACTTGCATACAGAC |
| BnUBC13D-F | CTCGGATCCAAATGGCTAATAGCAATCTACCG |
| BnUBC13D-R | CAGTGTCGACTCAAGCGCCACTTGCATATAGAC |
| BnUBC13E-F | CTCGGATCCAAATGGCCAACAGCAATCTTCCC |
| BnUBC13E-R | CAGTGTCGACTCATGCGCCGCTTGCGTACAGAC |
| BnUBC13F-F | CTCGGATCCAAATGGCGAATAGCAATCTACCG |
| BnUBC13F-R | CAGTGTCGACTCAAGCGCCACTTGCATATAGAC |
| BnUBC13G-F | CTCGGATCCAAATGGCGAATAGTAATCTTCCT C |
| BnUBC13G-R | CAGTGTCGACTCAAGCGCCACTTGCATATAGAC |
| BnUBC13H-F | CTCGGATCCAAATGGCCAACAGCAATCTGCCC |
| BnUBC13H-R | CAGTGTCGACTTATGCGCCGCTTGCGTACAGAC |
| BnUBC13I-F | CTCGGATCCAAATGGCCAACAGTAATCTCCC |
| BnUBC13I-R | CAGTGTCGACTCATTCGCCGCTTGCGTAAAGAC |
| BnUBC13J-F | CTCGGATCCAAATGGCCAACAGTAATCTACCC |
| BnUBC13J-R | CAGTGTCGACTCATGCACCGCTTGCGTAAAGAC |
| BnUBC13K-F | CTCGGATCCAAATGGCGAACAGTAGTCTACCA |
| BnUBc13K-R | CAGTGTCGACTTATGCGCCGCTGGCGTAAAGAC |
| BnUBC13L-F | CTCGGATCCAAATGGCCAACAGTAATCTCCCC |
| BnUBC13L-R | CAGTGTCGACTCATTCGCCGCTTGCGTAAAGAC |

**Supplementary figure legends**

**Fig. S1** A flowchart outlining experimental designs of this study.

**Fig. S2** Synteny analysis of *UBC13* family genes among *Arabidopsis thaliana*, *Brassica napus*, *B. rapa* and *B. oleracea*. Identified gene pairs were displayed by circus plots (<http://circos.ca/>). All chromosomes from four species are indicated by blocks scaled by chromosome length with chromosome names. The *UBC13* family genes and chromosomes from different species are indicated by different colors: red, *A. thaliana*; yellow, *B. napus*; purple, *B. rapa* and green, *B. oleracea*. Synteny gene pairs are linked by lines: pink, genes with a close relationship to *AtUBC13A*; brown, genes with a close relationship to *AtUBC13B*. For gene ID from *B. rapa*, the species name “_BraROA” is omitted to reduce the length for plotting.

**Fig. S3** Original images of (A) Fig. 6A and (B) Fig. 6B.

**Fig. S4** Original images of (A) Fig. 8A and (B) Fig. 8B.


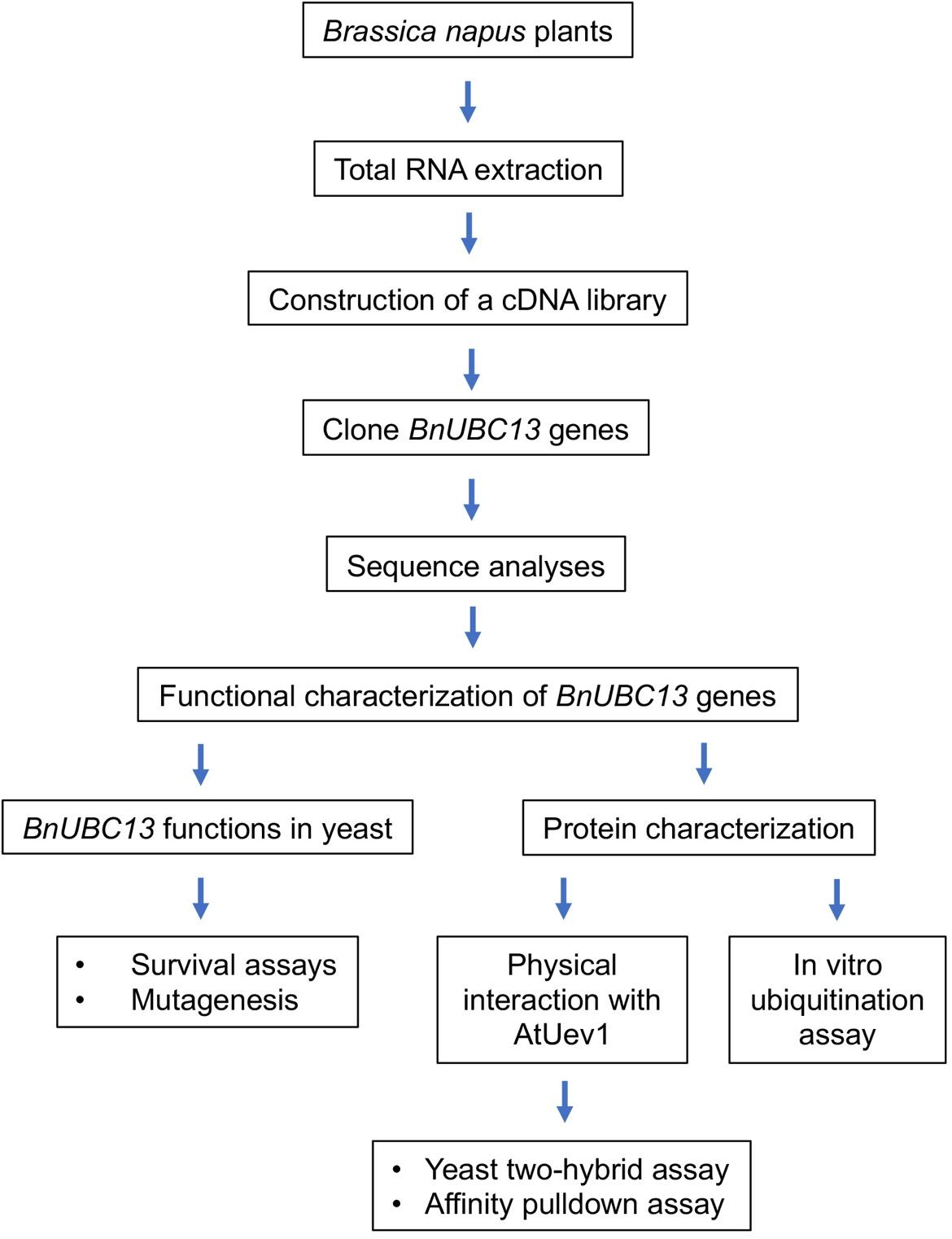


Figure S1


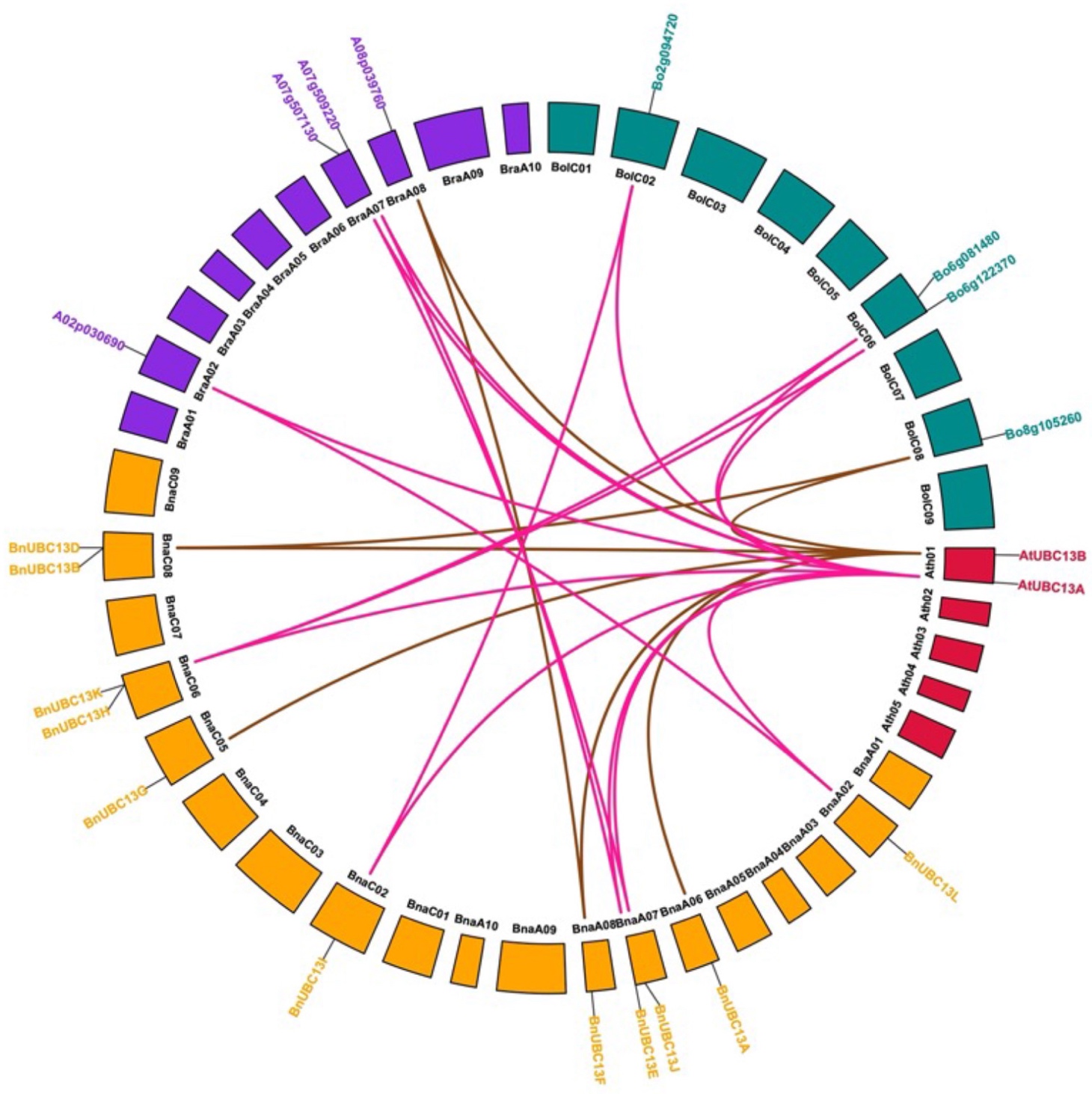


Figure S2


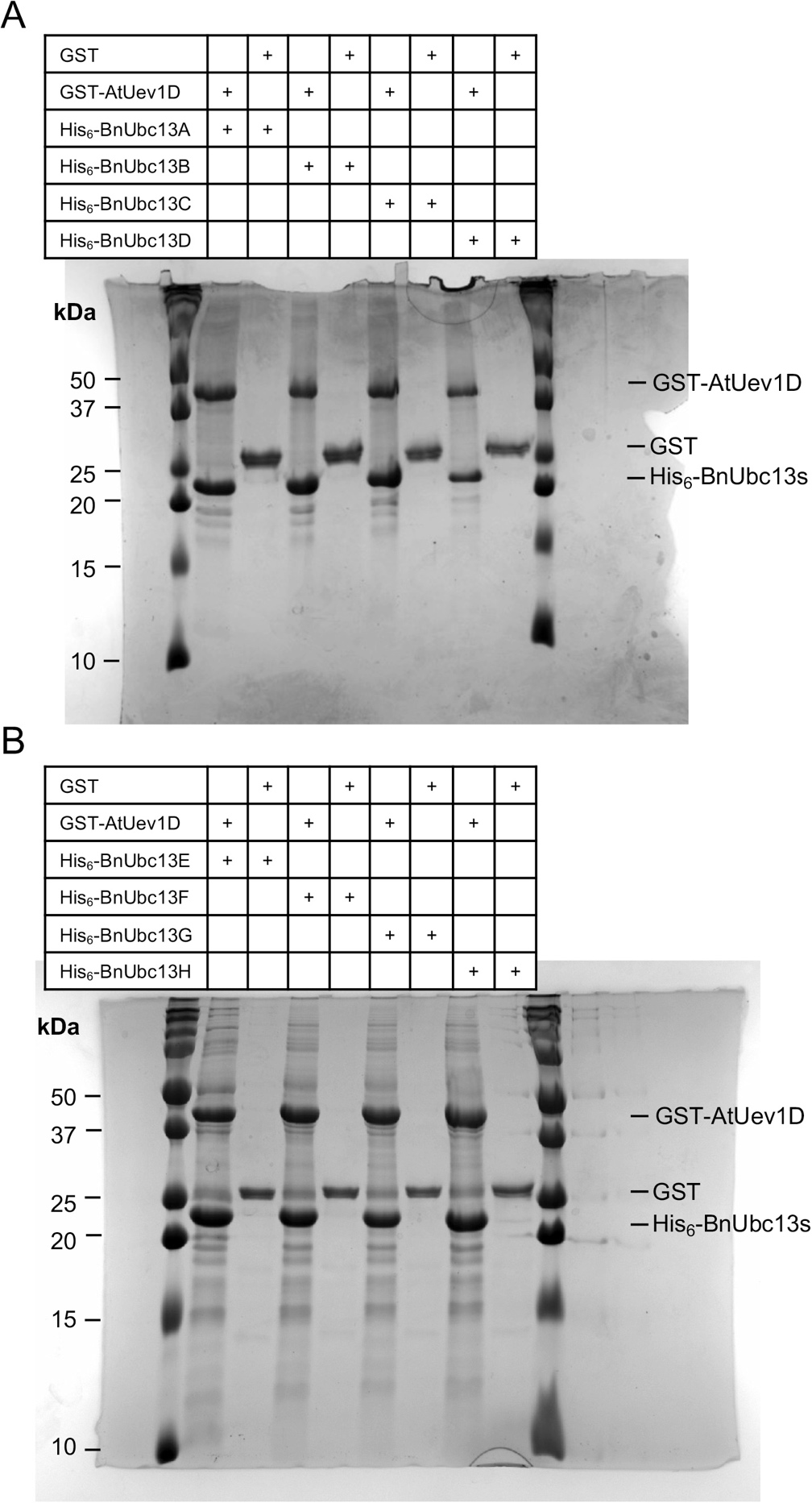


Figure S3


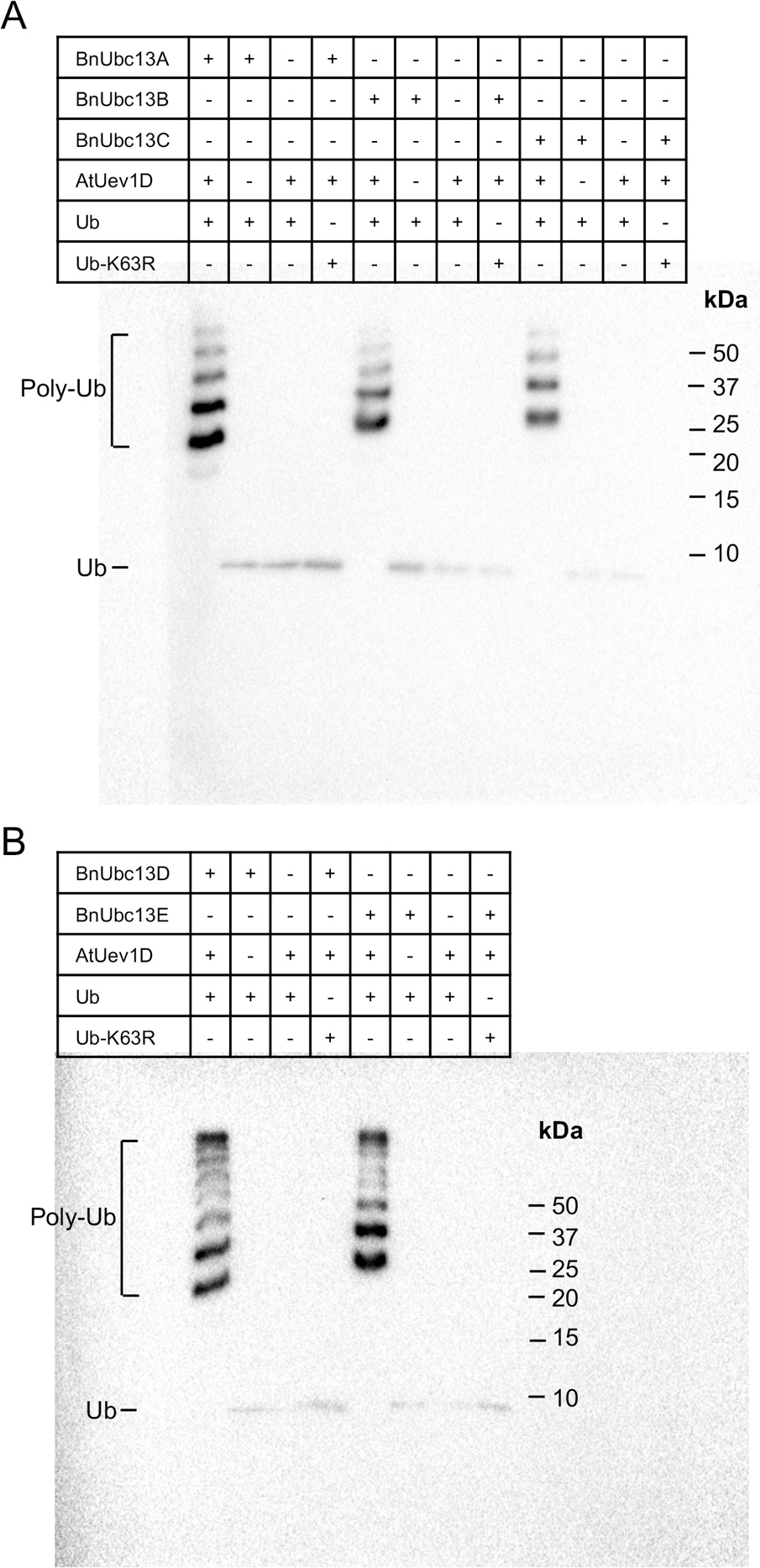


Figure S4
